# Developing a pharmacist-led intervention to provide transitional pharmaceutical care for hospital discharged patients: A collaboration between hospital and community pharmacists

**DOI:** 10.1016/j.rcsop.2022.100177

**Published:** 2022-09-05

**Authors:** Laura Victoria Jedig Lech, Charlotte Rossing, Trine Rune Høgh Andersen, Lotte Stig Nørgaard, Anna Birna Almarsdóttir

**Affiliations:** a Social and Clinical Pharmacy, Department of Pharmacy, University of Copenhagen, Universitetsparken 2, Copenhagen, Denmark; b Pharmakon, The Danish College of Pharmacy Practice, Milnersvej 42, 3400 Hillerød, Denmark; c Region Zealand Hospital Pharmacy, Clinical Pharmacy, Vestermarksvej 6, 4000 Roskilde, Denmark

**Keywords:** Transitional pharmaceutical care, Community pharmacy, Hospital pharmacy, Intraprofessional collaboration

## Abstract

**Background:**

Patients who transfer from the hospital back to the community are at risk of experiencing problems related to their medications. Hospital pharmacists (HPs) and community pharmacists (CPs) may play an important role and provide transitional pharmaceutical care in transition of care interventions.

**Objective:**

To describe how a pharmacist-led intervention to provide transitional pharmaceutical care for hospital discharged patients was developed, utilizing already existing pharmacist interventions in the hospital and community pharmacy.

**Methods:**

A mixed-method approach to intervention development was applied. Existing evidence was identified through a literature review of effective transitional care interventions and existing services in the hospital and community pharmacy. Focus group interviews and a workshop were carried out with HPs and CPs to identify their perceived facilitators and uncertainties in relation to intervention development. The final intervention and the expected outcomes were developed in an expert group workshop. Finally, the hospital part of the intervention was tested in a small-scale feasibility study to assess what type of information the HP would transfer to the CP for follow up.

**Results:**

Five components were identified through the 209 systematic reviews: pharmacist-led medication reconciliation, pharmacist-led medication review, collaboration with general practitioners (GPs), post discharge pharmacist follow up and patient counseling or education. HPs and CPs identified uncertainties related to the relevance of the information sent from the HP to the CP, identification of patients at the community pharmacy and communication. The expected outcomes for the patients receiving the intervention were an experience of increased safety and satisfaction and less use of healthcare resources. The feasibility study led to optimization of language and structure of the pharmacist referrals that were used to transfer information from the HP to the CP.

**Conclusion:**

A patient centered intervention to provide transitional pharmaceutical care for hospital discharged patients was developed using existing evidence in transition of care, HPs and CPs, an expert group, and a small-scale feasibility study. A full-scale feasibility test of the intervention should be carried out for it to be further refined.

## 1 Introduction

Transferring from one care setting of the healthcare system to another can be a high-risk process for the patient, and a successful transfer requires timely collaboration, coordination and information-sharing among healthcare professionals.^1^^,^^2^ Confirming that patients' medication lists are up to date and identifying and solving any problems related to their treatment is essential to decreasing the risk of medication errors and in turn, adverse drug events (ADEs) and unplanned use of healthcare resources.^3^^,^^4^ Patients transferring from the hospital to their own homes often have questions and doubts about their medications.^5^ These questions and doubts may lead to drug-related problems (DRPs) arising in the post-discharge period, creating sub-optimal treatment effects and an increased risk of readmission to hospital.^3^^,^^4^ Hospital pharmacists (HPs) and community pharmacists (CPs) identify and solve DRPs in various settings in the healthcare system in close collaboration with other healthcare professionals caring for the patient.^4^^,^^6^ The effect of pharmacist involvement in transitional care interventions has been well studied, but the results are heterogeneous due to the variety of outcome measures used to evaluate the effect of the interventions, poor descriptions of the usual care and the intervention, and bias.^1^^,^^7^, ^8^, ^9^, ^10^

Few studies have been carried out in primary care post-discharge, building upon collaborations between secondary and primary care or involving CPs.^11^, ^12^, ^13^, ^14^, ^15^ A majority of DRPs arise post-discharge, especially within the first 2 weeks, stressing the need for community-based pharmacists to take part in the transition of care interventions post-discharge.^16^^,^^17^ CPs' roles have mainly been explored in relation to following up on pending and unsolved DRPs from the hospital stay, identifying and solving DRPs in close collaboration with the patient's general practitioner (GP) and advising the patient on adherence and the optimal use of medications.^9^^,^^13^^,^^15^^,^^18^ Following up on identified but unsolved DRPs from the hospital stay may strengthen the impact of pharmacist interventions conducted at the hospital.^15^ Patients feel unsafe and are in high need of support and guidance right after discharge from hospital.^19^^,^^20^ Additionally, patients typically forget important information about changes to their drug treatments and are unable to ask the right questions at the point of discharge.^21^ Thus, there is a need to provide patients with a safe transition and readmission into primary care.

Healthcare utilization and low costs are important when developing a new intervention to increase the likelihood of it being cost-effective.^22^^,^^23^ Hence, it is important to explore how well-implemented and evidence-based pharmaceutical care services in the healthcare system can be combined and optimized to aid care transitions while securing optimal utilization of healthcare resources.

### 1.1 Aim

The aim of this study was to describe how a pharmacist-led intervention to provide transitional pharmaceutical care for hospital discharged patients was developed, utilizing already existing pharmacist interventions in the hospital and community pharmacy.

## 2 Ethics approval

The study was assessed by the regional ethical committee in Region Zealand, and it was concluded, that ethics approval was not required due to the nature of the study and the data that were gathered *(Reference number: 17–000048)*. Ethical considerations and precautions taken to protect human subjects in research relating to the focus group interviews are described in depth elsewhere.^26^ Prior to the two workshops all participants were informed about the project and which data would be used for scientific purposes. The participants were then asked to give an oral consent that the described data could be used in an anonymized format and published in a scientific journal. All patient data collected through the pilot study were gathered anonymously without any registration of personal patient data. The Danish Data Protection Agency at the Faculty of Health and Medical Sciences at the University of Copenhagen were consulted to ensure anonymous data collection in the pilot study.

## 3 Methods

A mixed-method approach was applied to develop the intervention, consisting of a review of the existing literature, focus group interviews, workshops, and a small-scale pilot. CPs and HPs were involved at various points during the intervention development (focus group interviews, workshop and pilot test) to provide their perspectives about the feasibility of its incorporation into the development process and to secure the integration of the new intervention into existing tasks in the hospital and the community pharmacy.^24^ The development of the intervention took place between March 2017 and October 2020.

An overview of the different methods used to develop the intervention and the aim related to each step (1–5) is depicted in Table 1.

**Table 1:** Schematic overview of the methods used in the development process of the new intervention.

Step	Method	Aim
1	Literature review	To identify existing evidence on pharmaceutical care interventions in care transitions locally and internationally.
2	Focus group interviews^25^	To identify CPs' and HPs' uncertainties and wishes toward the intervention.
3	CP and HP Workshop	To identify which tasks should be carried out as part of the new intervention and to identify uncertainties related to the new tasks.
4	Expert Group Workshop	To develop a preliminary version of the intervention based on steps 1–3 and identifying the expected outcomes of the intervention.
5	Feasibility study in hospital	To assess the ability of HPs to carry out new intervention tasks and allowing the HPs to generate experience prior to the full pilot test.

CP = Community Pharmacist, HP = Hospital Pharmacist.

### 3.1 Setting

The intervention was developed to be implemented in the Region Zealand, one of the five regions in Denmark, encompassing one regional hospital pharmacy and 34 community pharmacies. In Denmark, pharmacists employed at community pharmacies mainly carry out tasks related to dispensing, quality control and assurance, over-the-counter counseling, and, in recent years, pharmaceutical care services. The Region Zealand hospital pharmacy offers clinical pharmacy services to the wards of the eight hospitals in the region as well as three psychiatry centers. The HPs also carry out tasks related to the rational use of medications, hospital formularies, securing medication availability for the hospital wards, and medication counseling of healthcare professionals. The shared medication record (SMR) has been nationally implemented in Denmark to provide healthcare professionals with an overview of the patient's current list of prescribed medications. All healthcare professionals, including HPs and CPs, can access this platform with consent from the patient. SMR should be reconciled in care transitions,^26^^,^^27^ and thus the SMR needs to be updated at hospital discharge by a hospital doctor.

### 3.2 Literature review

PubMed was searched for systematic reviews that studied the effects of pharmacist interventions to optimize medication in care transitions. The search strategy was based on a Boolean search strategy where keywords and MeSH terms were combined related to the setting (transition of care) and the interventions (see Appendix A). The aim was to identify the type of interventions and the component of the interventions with a positive impact on clinical outcomes. Studies related to psychiatric patients specifically and patients discharged to other institutions, for example, nursing homes or palliative care, were excluded as the new intervention should target users able to visit the community pharmacy. Additionally, studies describing interventions related to one specific disease or patients being discharged from intensive care units were excluded.

Grey literature was screened to identify the structure and content of existing services related to medication optimization that were implemented in Region Zealand in the hospital and community pharmacies. This was carried out by reviewing manuals and, if relevant, published research for the implemented services from the involved pharmacists.

### 3.3 Focus group interviews

The methods and results from the focus groups are described elsewhere.^25^ Seven CPs and four HPs participated in the focus group interviews. The CPs and HPs were shown a preliminary version of the proposed intervention based on the identified evidence base. The results from the focus group interviews used to inform intervention development were extracted.

### 3.4 CP and HP workshop

A workshop was carried out with four HPs and four CPs already informed about the project. The HPs indicated interest in participating based on the focus group interview invitation. Two CPs continued from the focus group interviews, and two additional CPs were invited to participate, as their pharmacy owner showed interest based on the focus group interviews. The aim of the workshop was to identify tasks to be carried out as part of the new intervention and identifying uncertainties related to the new tasks. Besides the HPs and CPs, three of the authors (CR, LVJL, TRHA) facilitated the workshop. The workshop started with an introduction to the project background, aim, preliminary results from the focus group interviews, and a presentation of the intervention. Then, the workshop was divided into four workgroup sessions. The same group worked together in all workgroup sessions and consisted of one CP and one HP. A thorough description of the aim of each workgroup session can be seen in Appendix B. Each group took notes on paper that was collected at the end of the workshop. Additionally, facilitators took notes from each group during the plenary presentation and discussion. These notes were analyzed for emerging themes and suggestions for further developing the intervention.

### 3.5 Expert group workshop

An expert group comprising the four authors, representing both CPs, HPs, and academia, joined to specifically develop the community pharmacy part of the intervention, model all processes and discuss the expected outcomes of the intervention. The workshop session started with a summary of the literature review and key points from the focus group interviews and the CP and HP workshop. Based on the summary, the expert group developed the final version of the intervention, ready for the pilot test, and proposed which processes in the intervention would lead to the expected outcomes.

### 3.6 Feasibility test at the hospital

A small-scale feasibility study was carried out to test the new intervention-related tasks at the hospital. This was intended to identify any issues or problems related to the new tasks and to identify whether they were feasible alongside the normal work procedures. The feasibility study was carried out on the four acute wards in Region Zealand, where clinical pharmacy services (CPS) were already implemented. During their usual workflow at the acute ward, HPs practiced the new intervention tasks and produced referrals to be followed up post discharge. The referrals were analyzed in relation to the types of DRPs and classified using the Pharmaceutical Care Network Europe (PCNE) basic classification V6.2 ^28^ by LVJL. The PCNE classification was developed by Pharmaceutical Care Network Europe, as a tool used to describe the nature and type of DRPs identified through pharmacist interventions. The classification is divided into Problems (P-codes), Causes (C-codes), Interventions (I-codes), Acceptance of the intervention (A-codes) and Outcome of the intervention (O-codes). The referrals were classified on a basic level using only P, C, and I-codes. The A- and O-codes were irrelevant for this study, as the referrals were not actually followed up post-discharge. Using the basic classification in contrast to the more detailed sub-domain classification were deemed sufficient to grasp the overall type of the DRPs. The number of pharmacist referrals describing medication discrepancies, proposed medication changes and referrals directly to an existing pharmaceutical care intervention at the community pharmacy were counted and calculated as the percentages of all referrals. All data were gathered anonymously, without registration of any personal data. Three CPs who were to participate in the following feasibility study were asked to give feedback on three selected cases from the pharmacist referrals. The CPs discussed whether it was possible to use the pharmacist referral, follow up on the described DRPs post discharge, and suggested how the pharmacist referral structure and content could be optimized.

## 4 Results

### 4.1 Literature review

Through the literature review, 209 systematic reviews were identified, among which 18 were related to pharmacists' involvement in the transition of care.^8^, ^9^, ^10^, ^11^^,^^29^, ^30^, ^31^, ^32^, ^33^, ^34^, ^35^, ^36^, ^37^, ^38^, ^39^, ^40^, ^41^, ^42^ Three additional articles were identified through screening references included in the identified systematic reviews.^7^^,^^43^^,^^44^ The main outcome measures in the identified articles were 30-day readmissions, emergency department visits and length of stay. A few studies also measured the effect of the interventions on mortality.

#### 4.1.1 Evidence of the effective intervention components

Multi-faceted interventions with more components than a single intervention were identified as being more effective in reducing 30-day readmission rates.^11^^,^^30^^,^^33^^,^^34^^,^^43^, ^44^, ^45^ This effect increased with every pharmacologically intensive component added to the interventions that had both a hospital and community-based component.^11^ There was no homogenous evidence that CP involvement as a single component had any effect on readmissions. CPs should, however, be provided with essential information about the patient's hospital stay, collaborate with GPs, and take part in other intervention components. Table 2 summarizes the evidence supporting the intervention components described in the identified systematic reviews.

**Table 2:** Identified evidence on the intervention components among the hospital-discharge interventions.

Component	Evidence and related references
Pharmacist-led medication reconciliation	Heterogeneous evidence as a single intervention, but important as part of multi-faceted interventions, especially in combination with medication review, follow-up and patient counseling.^29^^,^^30^^,^^33^, ^34^, ^35^^,^^38^^,^^43^
Pharmacist-led medication review	Intensive pharmacological intervention component. As a stand-alone intervention it lacks evidence of any effects on mortality and readmissions. Can be combined with other intervention components that have an effect on readmissions. No evidence found for effectiveness when carried out post-discharge.^7^^,^^10^^,^^11^^,^^43^
Collaboration with GPs	Intensive pharmacological intervention component. Typically conducted as part of interventions with a medication review component. Collaboration at the point of discharge or post-discharge. Face-to-face communication is more effective than written communication in reducing readmissions.^31^^,^^33^^,^^43^
Post discharge pharmacist follow-up with the patient (by phone or home-visit)	No evidence for structured general follow-up by telephone. Should be combined with medication reconciliation, collaboration with GPs and should be tailored to patient's needs (e.g., with patient education/counseling as part of the follow-up).^32^^,^^33^
Patient counseling/Patient education	Intensive pharmacological intervention component that should be tailored to patient needs. Can be carried out during the hospital stay, at discharge or in the community.^11^^,^^30^^,^^32^^,^^37^^,^^43^

GP = General Practitioner.

#### 4.1.2 Evidence of implemented interventions in the community pharmacy

A review article was identified describing the five existing and implemented pharmaceutical care services in the community pharmacies in Denmark and the assembled evidence about these services.^46^ The adherence services for new medication and chronic medication users, as well as the inhaler technique assessment service (ITAS), are the most widely offered and implemented services, as they are fully reimbursed by the state.^46^, ^47^, ^48^ The adherence services are based on parts of the Safe and Effective Use of Medication (SEM) program tested in patients with high blood pressure, which has shown a significant increase in disease knowledge.^49^, ^50^, ^51^, ^52^ Neither of the interventions have been tested specifically in relation to hospital discharged patients.

#### 4.1.3 Evidence of implemented interventions in the hospital pharmacy

Two articles were identified describing the evidence about an intervention implemented in the acute wards in Region Zealand. The pharmacists are involved in clinical pharmacy services in the acute wards, where they conduct CPS when a patient is admitted to the hospital. Typically, patients are admitted through the acute ward of one of the four main hospitals, where they are assessed within 4 h and subsequently either discharged or admitted to an inpatient ward. The CPS includes a medication reconciliation and medication review aimed at optimizing the patient's medication safety and identifying and resolving DRPs.^6^^,^^53^ The identified DRPs are communicated to a hospital doctor through a pharmacist note in the patient journal, describing the problem and the suggested interventions. The service identified DRPs in 2/3 of patients^6^ and a reduction of medication-related harm during hospital stay by 50%, but with no effect on readmissions or mortality.^53^

### 4.2 Focus group interviews

In the focus group interviews, a preliminary version of the intervention based on the literature review was presented to the CPs and HPs.^25^ The preliminary version consisted of the already implemented service at hospital admission (CPS) and a follow up at the community pharmacy by a CP post discharge. The HPs shared DRPs identified as part of CPS at hospital admission with the CP post discharge. The CP could then follow up on and solve the transferred DRPs alongside identifying and solving new (if any) DRPs with the patient post discharge.

HPs and CPs provided valuable views on the limitations of the existing services, specifically CPS, that should be accounted for in the intervention. CPS is conducted at the time of admission and the status of the DRPs are unknown when the CPs need to follow up post discharge. A hospital doctor might already have assessed the relevant DRPs and implemented changes or deemed DRPs irrelevant. Additionally, the patient's health status or the drug treatment might have changed between admission and discharge. Since there is no intervention conducted to assess the status of the DRPs prior to discharge, the CPs need to conduct a medication reconciliation prior to patient follow up at the community pharmacy. This, however, conflicted with the CPs' wishes to offer the follow up on the spot, as was already done in the adherence services for new and chronic medication users. It should be further explored whether it is possible to combine on-the-spot follow up and medication reconciliation.^26^ The CPs wanted any follow up or new pharmaceutical care service at the community pharmacy to be structured like the adherence services for new and chronic medication users. Furthermore, it should be possible for the CPs to offer eligible patients any already existing and implemented pharmaceutical care services alongside carrying out the follow up.

Both CPs and HPs wished to be able to communicate on an ad hoc basis with one another about relevant patient cases. HPs could be provided with permission to access the electronic patient record post discharge to provide CPs with information related to medication changes and the reasons for them. However, relevant agreements should be made in relation to communication pathways and availability. All these data are described more thoroughly in the published article by Lech et al.^25^

### 4.3 HP and CP workshop

In the workshop, the CPs and HPs developed processes and tasks that needed to be carried out for the information from the HP to be received by the CP. The pharmacists identified the following new tasks in relation to the collaboration: Identification of patients suitable and information relevant for follow up at the community pharmacy, transferring information from HP to CP, receiving information at the community pharmacy and patient identification at the community pharmacy. Additionally, the CPs decided on the content and type of information transferred. Lastly, the CPs reflected on uncertainties related to the feasibility of the handover process.

DRPs could not be followed up at the community pharmacy and the CPs required additional information. It was decided to compile the information including the DRPs found at admission into a “Pharmacist Referral” and a template for this was developed (see Appendix C). Besides the information on DRPs, the following information was included on request from the CPs: Information about relevant drug-drug interactions, a reconciled medication list from the admission and the reason for admission, information on whether the patient had been informed about the referred DRPs, and compliance-related issues, if any.

The CPs and HPs agreed that an existing correspondence system could be used to send information (i.e., pharmacist referral from the HP to the CP). Healthcare professionals from different settings (i.e., the community, nursing homes, GP, hospital, and out-patient clinics in Denmark) can communicate through an encrypted communication form called “Correspondence messages”. However, the CP still needed to manually enter in the pharmacy system that a specific patient had a referral to notify the personnel at the pharmacy counter. The CPs stressed the importance of training and informing personnel to pay attention to any pop-up messages in the dispensing system to ensure that they identified eligible patients, and this was also identified as an important step in relation to the feasibility of the intervention.

CPs and HPs noted that the correspondence system was not suitable for direct contact between HPs and CPs. Instead, e-mail and telephone calls were preferred for post discharge communication. However, the CPs and HPs did stress that there are different opening hours between the community pharmacy and in the hospital. The acute wards are only staffed with HPs Monday-Friday from around 8:00–15:30, and patients usually visit the community pharmacy after opening hours or over the weekend.

### 4.4 Expert group workshop

The expert group compiled evidence and knowledge from the focus group interviews and the workshop with the CPs and HPs to adjust the preliminary intervention model presented in the focus group interviews. The expert group workshop specifically aimed at modeling the community pharmacy part of the intervention.

The structure of the patient-CP conversation was inspired by the adherence services,^46^ where a short initial conversation and a follow-up after 2–3 weeks were combined. However, an option for offering complex patients more in-depth conversations was introduced between the initial conversation and the follow-up. A complex patient could be a patient with insecurities about the drug treatment, many medication changes during the hospital stay and many DRPs described in the HP referral. The intervention was tailored to the patient's needs by identifying patient goals and expectations in relation to their medication post discharge and aiming to reach this goal in the ensuing conversations. Goal setting was inspired by the SEM projects and was carried out alongside identifying and solving DRPs as a part of pharmaceutical care.^49^ If necessary, the patient's GP was contacted with consent from the patient. The final conversation structure and the content of each conversation can be seen in Table 3.

**Table 3:** Conversation structure and content of the patient-CP conversation post discharge.

I: Initial conversation (Mandatory)	
At first visit/contact with the community pharmacy post discharge Carried out at the community pharmacy *Estimated time use:* 20 min	• Compare medication lists in pharmacist referral and shared medication record to identify changes prior to the conversation. • Obtain the patient history of the hospital stay and medication changes. • Identify patient expectations of the community pharmacy. • Identify the patient's goals at the community pharmacy. • Identify new (if any) DRPs and solve non-complex DRPs from pharmacist referral. • Refer to other pharmaceutical care services (if necessary). • Assess the need for additional conversations or follow-up. • Inform the patient about the plan for the next conversation.


(IIa): Additional conversation(s) (Not mandatory, CP assesses need)	
*2–4 weeks after the initial conversation* Carried out at the community pharmacy *Estimated time use:* 20–60 min	• Compare medication lists from the pharmacist referral and shared medication record (if not already carried out). • Inform the patient about the plan for the conversation. • Identify new (if any) DRPs and solve pending DRPs from the pharmacist referral (if still relevant). • Revisit the patient goals, carry out interventions to reach the patient's goal(s), and assess the effects of the planned interventions. • Refer to other pharmaceutical care services (if necessary). • Assess the need for more conversations or follow-up. • Inform the patient about the aim of the next conversation.

IIb: Follow up conversation (Mandatory)	
2–4 weeks after the last conversation or initial conversation Via telephone *Estimated time use:* 10 min	• Inform the patient about the plan for the conversation. • Identify new (if any) DRPs by revisiting the goals. • Assess the effects of the earlier planned interventions and goal completion. • Assess the need for additional conversations if required or for completion of the intervention.

DRP = Drug-related problem, CP = Community Pharmacist.

**Fig. 1:**
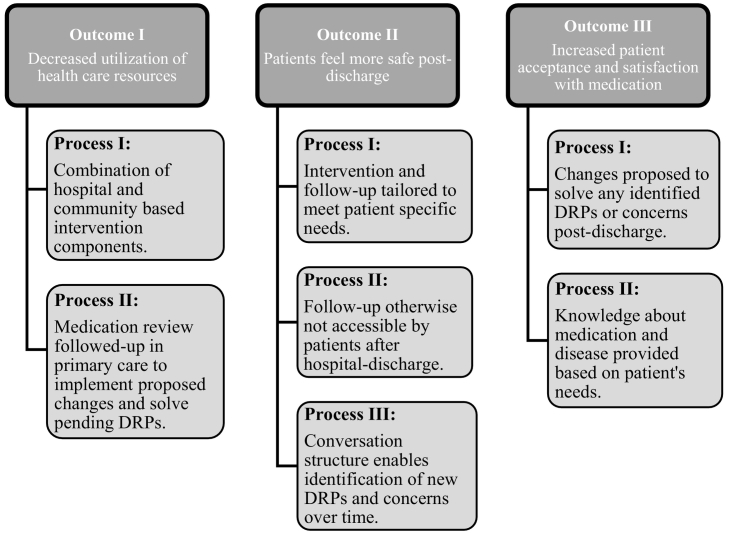
Expected outcomes of the developed intervention and the processes implemented in the intervention structure leading to these outcomes. DRPs = Drug-related problems.

At the end of the expert group workshop, the processes and parts of the intervention were modeled, relating the new processes to the expected outcomes (see Fig. 1).

### 4.5 Feasibility at the hospital

Eleven HPs participated across the four wards. For the relevant patients, the HP filled in the referral template (see Appendix C). Over the course of seven months (August 2019–March 2020), the HPs produced 131 pharmacist referrals. The HPs were able to identify a variety of DRPs to be followed up post discharge. The referrals were evenly produced by the four participating acute wards. The pharmacist referrals described a total of 227 DRPs yielding an average of 1.73 [1–8] DRPs. The DRPs were almost evenly distributed between P1*(treatment effectiveness*) (*n* = 104; 45.8%) and P2 (*treatment safety*) (*n* = 113; 49.8%). The problems were C7 (*patient-related*) in most cases (*n* = 74; 32.6%) but were also related to either C1 (*drug selection*) (*n* = 66; 29.1%) or C3 *(dose selection)* (*n* = 36; 15.9%).

The HPs suggested changes to the drug treatment that the CP could follow up on for half of the patients (*n* = 63; 48.1%). However, almost all DRPs were planned at I2 (*patient level*) (*n* = 202; 89.0%). The HPs identified medication discrepancies among 23.6% of all patients (*n* = 31). Additionally, HPs referred the patients directly to an existing pharmaceutical care intervention at the community pharmacy for 18.3% of the patients (*n* = 24).

CPs were asked to give feedback on three selected cases from the pharmacist referrals. The CPs deemed all three referrals relevant to be followed up at the community pharmacy but asked for a more fixed structure with headlines for longer referrals (e.g., with many DRPs) and a suggestion to write some clinical terms and sentences in layperson's language for better understanding. Additionally, patient information was highly valued, as also stated in the workshop with the CPs and HPs.

### 4.6 The resulting intervention

**Fig. 2:**
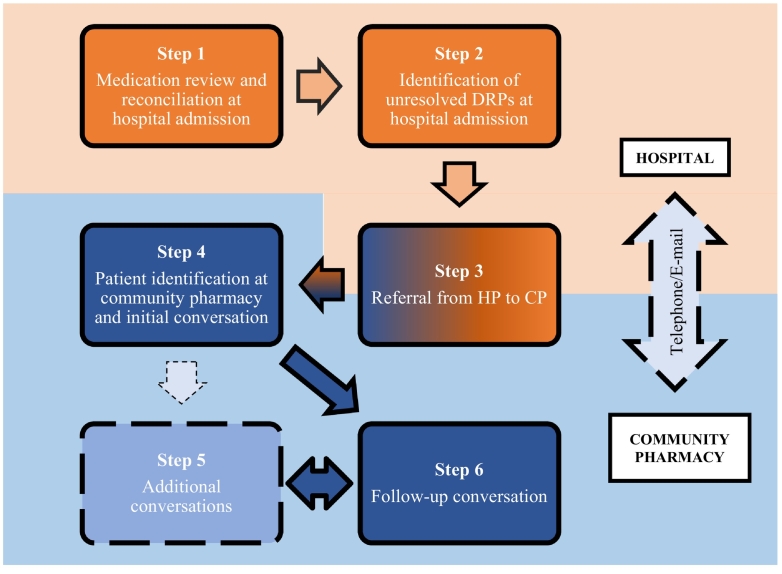
Stepwise process of the new pharmacist-led intervention to provide transitional pharmaceutical care for hospital-discharged patients. CP = Community Pharmacist, HP = Hospital Pharmacist, DRP = Drug-related problem.

The final intervention consists of three components (Fig. 2):

1) Medication review and reconciliation upon admission to the hospital, including the identification of DRPs (steps 1–2).
2) Referral from the hospital pharmacist to a community pharmacist (step 3).
3) Post discharge conversation at the community pharmacy (steps 4–6).

Additionally, the intervention consisted of an intra-professional collaboration component, where CPs and HPs are encouraged to communicate throughout the intervention (arrow between the hospital and community pharmacy).

## 5 Discussion

In this study, the development of a pharmacist-led intervention to provide transitional pharmaceutical care for hospital-discharged patients was described. CPs and HPs participated in the development of the intervention.

The developed intervention was based on existing and well-implemented services in both hospital and community pharmacies. A service consisting of medication reconciliation and a medication review was utilized to identify DRPs from the hospital admission for the CPs to follow up on post discharge. An option was added so the CPs could offer complex patients more conversations at the community pharmacy and conduct more interventions such as a medication review, an actual medication reconciliation, or in-depth patient education. Recognizing that not all patients require the same intervention or can handle the same amount of information at once, is important to keep the intervention patient-centered and based on patient needs.^21^^,^^43^^,^^54^ Few studies have tested the effects of multiple conversation structured post discharge interventions in relation to transition of care,^55^ and post discharge interventions at the community pharmacy have mainly been limited to a single conversation.^9^^,^^15^

This intervention structure also had some limitations. Because the intervention was carried out at only two times, at admission and post discharge, the CPs were unable to easily obtain an overview of the patient's hospital stay and did not know when the patients were discharged from hospital. CPs were only able to retrieve this information through a phone call to the HP, who could look up information in the patient's journal. These insights are important for the CP to be able to have the most optimal role in care transitions^9^^,^^43^ and may jeopardize the expected outcomes of the intervention. Although patients typically get prescribed new drugs or need to pick up drugs post discharge, it is unknown when the first planned visit to the community pharmacy will be. As a majority of ADEs happen in the first 14 days post discharge, patients might be experiencing an ADE or a DRP that remains unsolved for many days post discharge.^16^

The development process described in this study had both strengths and limitations. CPs and HPs were involved throughout the development and mainly supplemented the intervention with knowledge related to the limitations and possibilities of the existing structures and systems, and thus strongly influenced the intervention setup. Practical problems and the ability of an intervention to fit into current work structures and procedures is of utmost importance for the feasibility of the intervention.^24^ The development process was carried out using a pragmatic mixed-methods approach, utilizing the ability of different methods to capture and assist in the development of specific parts of the intervention. The intervention was developed through existing evidence, engagement of CPs and HPs and considerations of the context for implementation. Additionally, CPs and HPs were able to share their key uncertainties during the intervention development. These aspects are core elements in intervention development in the new guideline on developing complex interventions by the Medical Research Council (MRC).^22^

Even though the development process did align with some suggestions proposed by the MRC guideline, such as engaging stakeholders, considering the context and identifying key uncertainties, the intervention development was not theory-based and was based on a pragmatic approach.^22^^,^^23^ MRC highly recommends that intervention development be based on an existing or identified theory for the intervention to have any effects on the intended outcomes. The development of the intervention did not involve other relevant stakeholders, such as patients and GPs. It is clear that supplementing the development with input from other stakeholders or directly co-designing it with patients might have changed the intervention design.^22^^,^^23^ Additionally, GPs play an active and important role in relation to implementing any necessary changes in the post discharge follow up.^25^^,^^31^

The pilot study that was carried out provided valuable insight into the type of DRPs chosen by HPs for follow-up by the CP. For example, HPs suggested changes to the drug treatment in almost half of the referrals, highlighting the need for CPs to assess which DRPs are still relevant through medication reconciliation post discharge. It was deemed feasible based on the pilot study for HPs to identify relevant DRPs that are suitable for follow-up post discharge.

The next step is to feasibility test the full intervention to see how the handover from the HP to the CP works in practice, and whether the CPs in the current procedure can follow up and identify the patients after hospital discharge. The feasibility test should additionally shed light on how the CPs carry out the community pharmacy part of the intervention, especially as to which patients are offered more conversations than the initial conversation and a follow-up conversation and why.

## 6 Conclusion

A patient centered intervention to provide transitional pharmaceutical care for hospital discharged patients was developed using existing evidence in transition of care, HPs and CPs, an expert group, and a small-scale feasibility study. The resulting intervention consisted of an already implemented medication review and medication reconciliation at hospital admission by a HP, a series of patient centered conversations at the community pharmacy post discharge, collaboration between CPs and HPs using a referral template, e-mail and telephone and ad hoc collaboration with the patients' GP. A full-scale feasibility test of the intervention should be carried out for it to be further refined.

## Funding

This study was funded by 10.13039/501100005860Helsefonden. The study is part of a larger project, that has been funded by the Danish Federation of Community Pharmacies, The department of Pharmacy at the University of Copenhagen and the Hospital Pharmacies' and AMGROS' research and development fund. None of the funding partners participated in any aspects of the study, i.e., the study design, data collection, data analysis, interpretation of data, writing the report or the decision to publish the report.

## Declaration of Competing Interest

The authors declare that they have no known competing financial interests or personal relationships that could have appeared to influence the work reported in this paper.

## Figures and Tables

**Table:** 

SESSION NO.	AIM
Groupwork I	Concerns Each group picked out three of the most important concerns and a facilitator wrote on Post-its to remind participants and facilitators about the concerns throughout the workshop.
Groupwork II	Picking DRPs for transfer Each group discussed which DRPs could be chosen for follow-up by the CP and why. Additionally, what information was needed for the CPs to be able to follow up on the DRPs was discussed. The groups were presented with an inspiration paper giving examples of additional information that could be transferred besides the DRPs. This document was inspired by existing literature about what CPs are deemed as important information to have for patients post-discharge (x).
Groupwork III	Referral to CP Each group discussed how the DRPs should now be transferred to the CP for follow-up, whether it should be possible for the CP and HP to contact each other, and in which way it should be possible to contact each other.
Groupwork IV	Patient follow-up at the community pharmacy Each group discussed how the patient could be identified after discharge when the patient would visit the community pharmacy. Additionally, it was discussed whether there should be feedback to the HP.

**Table:** 

REFERRAL TEMPLATE	
Hospital pharmacist initials *Enter your initials.*	
Hospital admission cause *Specify the admission cause according to the patient.*	
Medication administration help *Specify whether the patient receives any help with medication administration.* *If YES, specify which type of help.*	
Reconciled medication list at admission *Copy actual medication list for the patient.* *Specify if there are medication discrepancies.*	
PHARMACIST REFERRAL	
Summary of the medication review *Describe the important aspects relevant to the community pharmacist about the medication review you conducted.*	
Message for the community pharmacist *Describe which aspects of the drug treatment the community pharmacist should follow up on with the patient.*	
SUPPLEMENTARY INFORMATION	
Changes to the drug treatment *Have the suggested changes to the drug treatment/follow-up areas been discussed with the patient? (Yes/No)*	
Interactions *Specify whether the medication review included screening for interactions and enter the clinically relevant interactions (if any).*	
Kidney disease *Does the patient have a history of chronic kidney disease?* *If yes, enter to which degree.*	
Liver disease *Does the patient have a history of chronic liver disease?* *If yes, enter to which degree.*	

## Appendix A Search strategy for identifying systematic reviews

((((“patient discharge”[MeSH Terms] OR “discharge”[Title/Abstract] OR “discharged”[Title/Abstract] OR “post discharge”[Title/Abstract] OR “postdischarge”[Title/Abstract] OR “hospitals”[MeSH Terms] OR “hospital setting”[Title/Abstract]) AND (“patient education as topic”[MeSH Terms] OR “counseling”[MeSH Terms] OR “counseling”[Title/Abstract] OR “medication counseling”[Title/Abstract] OR “knowledge”[Title/Abstract] OR “drug knowledge”[Title/Abstract] OR “medicines knowledge”[Title/Abstract] OR “medication knowledge”[Title/Abstract] OR “education”[Title/Abstract] OR “medication understanding”[Title/Abstract] OR “Medication Therapy Management”[MeSH Terms] OR “integrated medicines management” [Title/Abstract] OR “medicine management”[Title/Abstract] OR “drug management”[Title/Abstract] OR “Medication Errors”[MeSH Terms] OR “medication reconciliation”[MeSH Terms] OR “medication reconciliation”[Title/Abstract] OR “medical history taking”[MeSH Terms] OR “medication history taking”[Title/Abstract] OR “medication history”[Title/Abstract] OR “continuity of patient care”[MeSH Terms] OR “Patient Care Planning”[MeSH Terms] OR “discharge planning” [Title/Abstract] OR “discharge service”[Title/Abstract] OR “follow-up”[Title/Abstract] OR “follow-up”[Title/Abstract] OR “transition*”[Title/Abstract] OR “seamless”[Title/Abstract] OR “care bundle”[Title/Abstract] OR “care coordination”[Title/Abstract] OR “aftercare”[MeSH Terms] OR “aftercare”[Title/Abstract] OR “continuity”[Title/Abstract] OR “outreach”[Title/Abstract] OR “co-ordination”[Title/Abstract] OR “coordination”[Title/Abstract] OR “house calls”[MeSH Terms] OR “home visit*”[Title/Abstract] OR “house visit*”[Title/Abstract] OR “pharmacy visit”[Title/Abstract] OR “pharmacist visit”[Title/Abstract] OR “home based intervention”[Title/Abstract] OR “telephone call*”[Title/Abstract] OR “telephone”[Title/Abstract] OR “phone call*”[Title/Abstract] OR “phone”[Title/Abstract] OR “medication review”[Text Word] OR “drug utilization review”[MeSH Terms] OR “treatment review”[Title/Abstract] OR “medication review”[Title/Abstract] OR “medicines review”[Title/Abstract] OR “drug review”[Title/Abstract] OR “reviewing medication”[Title/Abstract] OR “monitoring pharmacotherapy”[Title/Abstract] OR “optimizing drug regimens”[Title/Abstract] OR “pharmaceutical care”[Title/Abstract]) AND (“community pharmacy services”[MeSH Terms] OR “pharmacy service, hospital”[MeSH Terms] OR “pharmaceutical preparations”[MeSH Terms] OR “drug prescriptions”[MeSH Terms] OR “pharmacists”[All Fields] OR “pharmacist*”[All Fields] OR “pharmacists”[MeSH Terms] OR “pharmacy”[Title/Abstract] OR “pharmacist”[Title/Abstract] OR “pharmacists”[Title/Abstract] OR “pharmaceutical”[Title/Abstract] OR “pharmacists aides”[Title/Abstract] OR “pharmacy technician”[Title/Abstract] OR “pharmacy practitioners”[Title/Abstract] OR “pharmacy service”[Title/Abstract] OR “medication”[Title/Abstract] OR “community pharmacy”[Title/Abstract])) NOT “admission”[Title/Abstract]) NOT “hospital admission”[Title/Abstract]) AND (systematicreview[Filter])

## References

[1] Hesselink G., Zegers M., Vernooij-Dassen M. (2014). Improving patient discharge and reducing hospital readmissions by using intervention mapping. BMC Health Serv Res.

[2] Cornish P.L., Knowles S.R., Marchesano R. (2005). Unintended medication discrepancies at the time of hospital admission. Arch Intern Med.

[3] Paulino E.I., Bouvy M.L., Gastelurrutia M.A., Guerreiro M., Buurma H. (2004). ESCP-SIR Rejkjavik community pharmacy research group. Drug related problems identified by European community pharmacists in patients discharged from hospital. Pharm World Sci.

[4] Ensing H.T., Koster E.S., van Berkel P.I., van Dooren A.A., Bouvy M.L. (2017). Problems with continuity of care identified by community pharmacists post-discharge. J Clin Pharm Ther.

[5] Hesselink G., Flink M., Olsson M. (2012). Are patients discharged with care? A qualitative study of perceptions and experiences of patients, family members and care providers. BMJ Qual Saf.

[6] Nielsen T.R.H., Andersen S.E., Rasmussen M., Honore P.H. (2013). Clinical pharmacist service in the acute ward. Int J Clin Pharm.

[7] Christensen M., Lundh A. (2016). Medication review in hospitalised patients to reduce morbidity and mortality. Cochrane Database Syst Rev.

[8] Lussier M.E., Evans H.J., Wright E.A., Gionfriddo M.R. (2020). The impact of community pharmacist involvement on transitions of care: a systematic review and meta-analysis. J Am Pharm Assoc.

[9] Nazar H., Nazar Z., Portlock J., Todd A., Slight S.P. (2015). A systematic review of the role of community pharmacies in improving the transition from secondary to primary care. Br J Clin Pharmacol.

[10] Graabaek T., Kjeldsen L.J. (2013). Medication reviews by clinical pharmacists at hospitals lead to improved patient outcomes: a systematic review. Basic Clin Pharmacol Toxicol.

[11] Daliri S., Boujarfi S., El Mokaddam A. (2021). Medication-related interventions delivered both in hospital and following discharge: a systematic review and meta-analysis. BMJ Qual Saf.

[12] Tetuan C.E., Guthrie K.D., Stoner S.C., May J.R., Hartwig D.M., Liu Y. (2018). Impact of community pharmacist-performed post-discharge medication reviews in transitions of care. J Am Pharm Assoc.

[13] Daliri S., Hugtenburg J.G., Ter Riet G. (2019). The effect of a pharmacy-led transitional care program on medication-related problems post-discharge: a before-after prospective study. PLoS One.

[14] Cossette B., Ricard G., Poirier R. (2020). Pharmacist-led transitions of care for older adults at risk of drug-related problems: a feasibility study. Res Social Adm Pharm.

[15] Ensing H.T., Koster E.S., Dubero D.J., van Dooren A.A., Bouvy M.L. (2019). Collaboration between hospital and community pharmacists to address drug-related problems: the HomeCoMe-program. Res Social Adm Pharm..

[16] Kanaan A.O., Donovan J.L., Duchin N.P. (2013). Adverse drug events after hospital discharge in older adults: types, severity, and involvement of beers criteria medications. J Am Geriatr.

[17] Ensing H.T., Koster E.S., Stuijt C.C.M., van Dooren A.A., Bouvy M.L. (2015). Bridging the gap between hospital and primary care: the pharmacist home visit. Int J Clin Pharm.

[18] Brühwiler L.D., Hersberger K.E., Lutters M. (2017). Hospital discharge: what are the problems, information needs and objectives of community pharmacists? A mixed method approach. Pharm Pract (Granada)..

[19] Schultz H., Lundby C., Filipsen J., Rasmussen S., Pottegård A. (2019). Surgical patients’ experiences of information about medication: a qualitative comparative study with a patient-centered medication counseling upon discharge. Eur J Pers Cent Healthc.

[20] Hanssen T.A., Nordrehaug J.E., Hanestad B.R. (2005). A qualitative study of the information needs of acute myocardial infarction patients, and their preferences for follow-up contact after discharge. Eur J Cardiovasc Nurs.

[21] Eibergen L., Janssen M.J.A., Blom L., Karapinar-Çarkit F. (2018). Informational needs and recall of in-hospital medication changes of recently discharged patients. Res Social Adm Pharm..

[22] Skivington K., Matthews L., Simpson S.A. (2021). A new framework for developing and evaluating complex interventions: update of Medical Research Council guidance. BMJ (Clinical research ed).

[23] O’Cathain A., Croot L., Duncan E. (2019). Guidance on how to develop complex interventions to improve health and healthcare. BMJ Open.

[24] Bowen D.J., Kreuter M., Spring B. (2009). How we design feasibility studies. Am J Prev Med.

[25] Lech L.V.J., Husted G.R., Almarsdottír A.B., Andersen T.R.H., Rossing C., Nørgaard L.S. (2020). Hospital and community pharmacists’ views of and perspectives on the establishment of an intraprofessional collaboration in the transition of care for newly discharged patients. Innov Pharm.

[26] Fælles Medicinkort (FMK) (2021). Sundhedsdatastyrelsen. https://sundhedsdatastyrelsen.dk/da/registre-og-services/om-faelles-medicinkort.

[27] Andersen T.S., Gemmer M.N., Sejberg H.R. (2022). Medicines reconciliation in the emergency department: important prescribing discrepancies between the shared medication record and patients’ actual use of medication. Pharmaceuticals..

[28] Pharmaceutical Care Network Europe Foundation (2010). The PCNE Classification V 6.2. Pharmaceutical Care Network Europe. http://www.pcne.org/upload/files/11_PCNE_classification_V6-2.pdf.

[29] McNab D., Bowie P., Ross A., MacWalter G., Ryan M., Morrison J. (2018). Systematic review and meta-analysis of the effectiveness of pharmacist-led medication reconciliation in the community after hospital discharge. BMJ Qual Saf.

[30] Kwan J.L., Lo L., Sampson M., Shojania K.G. (2013). Medication reconciliation during transitions of care as a patient safety strategy: a systematic review. Ann Intern Med.

[31] Foot H., Scott I., Sturman N. (2022). Impact of pharmacist and physician collaborations in primary care on reducing readmission to hospital: a systematic review and meta-analysis. Res Social Adm Pharm..

[32] Capiau A., Foubert K., Van der Linden L. (2020). Medication counselling in older patients prior to hospital discharge: a systematic review. Drugs Aging.

[33] Hesselink G., Schoonhoven L., Barach P. (2012). Improving patient handovers from hospital to primary care: a systematic review. Ann Intern Med.

[34] Tomlinson J., Cheong V.L., Fylan B. (2020). Successful care transitions for older people: a systematic review and meta-analysis of the effects of interventions that support medication continuity. Age Ageing.

[35] Domingo G.R.R., Reyes F.C., Thompson F.V., Johnson P.M., Shortridge-Baggett L.M. (2012). Effectiveness of structured discharge process in reducing hospital readmission of adult patients with community acquired pneumonia: a systematic review. JBI Libr Syst Rev.

[36] Bethishou L., Herzik K., Fang N., Abdo C., Tomaszewski D.M. (2020). The impact of the pharmacist on continuity of care during transitions of care: a systematic review. J Am Pharm Assoc.

[37] Bonetti A.F., Reis W.C., Mendes A.M. (2020). Impact of pharmacist-led discharge counseling on hospital readmission and emergency department visits: a systematic review and meta-analysis. J Hosp Med.

[38] Mekonnen A.B., McLachlan A.J., Brien J.A.E. (2016). Effectiveness of pharmacist-led medication reconciliation programmes on clinical outcomes at hospital transitions: a systematic review and meta-analysis. BMJ Open.

[39] Kooyman C.D.A., Witry M.J. (2019). The developing role of community pharmacists in facilitating care transitions: a systematic review. J Am Pharm Assoc.

[40] Bahr S.J., Solverson S., Schlidt A., Hack D., Smith J.L., Ryan P. (2014). Integrated literature review of postdischarge telephone calls. West J Nurs Res.

[41] van Loon-van Gaalen M., van Winsen B., van der Linden M.C., Gussekloo J., van der Mast R.C. (2021). The effect of a telephone follow-up call for older patients, discharged home from the emergency department on health-related outcomes: a systematic review of controlled studies. Int J Emerg Med.

[42] Hansen L.O., Young R.S., Hinami K., Leung A., Williams M.V. (2011). Interventions to reduce 30-day rehospitalization: a systematic review. Ann Intern Med.

[43] Ensing H.T., Stuijt C.C.M., van den Bemt B.J.F. (2015). Identifying the optimal role for pharmacists in care transitions: a systematic review. J Manag Care Spec Pharm.

[44] Skjot-Arkil H., Lundby C., Kjeldsen L.J. (2018). Multifaceted pharmacist-led interventions in the hospital setting: a systematic review. Basic Clin Pharmacol Toxicol..

[45] Rodrigues C.R., Harrington A.R., Murdock N. (2017). Effect of pharmacy-supported transition-of-care interventions on 30-day readmissions: a systematic review and meta-analysis. Ann Pharmacother.

[46] Abrahamsen B., Burghle A.H., Rossing C. (2020). Pharmaceutical care services available in Danish community pharmacies. Int J Clin Pharm.

[47] Herborg H., Soendergaard B., Froekjaer B. (2001). Improving drug therapy for patients with asthma--part 1: Patient outcomes. J Am Pharm Assoc (Wash).

[48] Herborg H. (2001). Improving drug therapy for patients with asthma-part 2: use of antiasthma medications. J Am Pharm Assoc (Wash)..

[49] Kjeldsen L.J., Bjerrum L., Dam P. (2015). Safe and effective use of medicines for patients with type 2 diabetes - a randomized controlled trial of two interventions delivered by local pharmacies. Res Social Adm Pharm..

[50] Kaae S., Dam P., Rossing C. (2016). Evaluation of a pharmacy service helping patients to get a good start in taking their new medications for chronic diseases. Res Social Adm Pharm..

[51] Herborg H. (2015). Safe and effective use of medicines for ethnic minorities - a pharmacist-delivered counseling program that improves adherence. J Pharma Care Health Sys.

[52] Herborg H., Haugbølle L.S., Sørensen L., Rossing C., Dam P. (2008). Developing a generic, individualised adherence programme for chronic medication users. Pharm Pract (Granada).

[53] Nielsen T.R.H., Honoré P.H., Rasmussen M., Andersen S.E. (2017). Clinical effects of a pharmacist intervention in acute wards - a randomized controlled trial. Basic Clin Pharmacol Toxicol..

[54] Berendsen A.J., de Jong G.M., Meyboom-de Jong B., Dekker J.H., Schuling J. (2009). Transition of care: experiences and preferences of patients across the primary/secondary interface - a qualitative study. BMC Health Serv Res.

[55] Ravn-Nielsen L.V., Duckert M.L., Lund M.L. (2018). Effect of an in-hospital multifaceted clinical pharmacist intervention on the risk of readmission: a randomized clinical trial. JAMA Intern Med.

